# Clinical and imaging findings of patients diagnosed with adenovirus-positive pneumonia during 2015–2019 in Shanghai, China

**DOI:** 10.3906/sag-2010-132

**Published:** 2021-10-30

**Authors:** Chunrong HUANG, Dong WEI, Yahui LIU, Guochao SHI

**Affiliations:** 1 Department of Pulmonary and Critical Care Medicine, Ruijin Hospital, Shanghai Jiao Tong University School of Medicine, Shanghai, People’s Republic of China; 2 Institute of Respiratory Diseases, Shanghai Jiao Tong University School of Medicine, Shanghai, People’s Republic of China; 3 Research Laboratory of Clinical Virology, Ruijin Hospital, Shanghai Jiaotong University School of Medicine, Shanghai, People’s Republic of China

**Keywords:** Adenovirus, pneumonia, chest CT scan, T cells

## Abstract

**Background/aim:**

This study was to describe the clinical characteristics, chest CT image findings, and potential role of T cells immunity in adenovirus positive pneumonia.

**Materials/methods:**

In this retrospective study, medical records of 53 adult Adv+ patients who were admitted to the Ruijin Hospital, Shanghai Jiao Tong University School of Medicine, from May 2015 to August 2019 were included. The presence of adenovirus and other respiratory viruses was detected using polymerase chain reaction of throat swabs samples. Clinical features and chest computed tomography (CT) findings were compared between patients with Adv+ pneumonia and Adv+ non-pneumonia.

**Results:**

The top 3 most commonly occurring symptoms in Adv+ pneumonia patients were fever (66.7%), cough (63.3%), and tachypnea (16.7%). Patients with Adv+ pneumonia showed significantly higher rates of cough and fever and longer duration of hospitalization than patients with Adv+ non-pneumonia. In the Adv+ pneumonia group, consolidation (73.3%) was the most common imaging finding on chest CT scan, and the likelihood of involvement of bilateral lobes (60%) was high. Classical conspicuous consolidation with surrounding ground-glass opacity was observed in 5 (16.6%) patients with Adv+ pneumonia. Patients with Adv+ pneumonia showed a higher inhibition of T-cell immunity than did patients with Adv+ non-pneumonia, and counts of CD3^+^, CD4^+^, and CD8^+^ T-cells may predict the presence of pneumonia in Adv+ patients.

**Conclusion:**

With regard to Adv+ pneumonia, the most frequent symptoms were cough and fever, and the most common CT pattern was consolidation; classical CT findings such as consolidation with surrounding ground-glass opacity could also be observed. Furthermore, our data indicated the incidence of abrogated cellular immunity in patients with Adv+ pneumonia.

## 1. Introduction

Adenovirus (Adv), a non-enveloped double-stranded DNA virus, is quite a common contagious pathogen that causes upper or lower respiratory tract infections in neonates, children, and adults, especially in those with compromised immunity, such as HIV-positive individuals or patients who have undergone transplantation [1–3]. In immunocompetent subjects, adenovirus generally causes mild infections, which are usually self-limiting; however, the incidence of severe pneumonia or acute respiratory distress syndrome (ARDS), for which virulence of newly emergent adenovirus, immune incompetence, or irregulated host immune responses may serve as underlying mechanisms, has also been reported in some studies.

In recent decades, partly owing to several outbreaks of adenovirus infections, such as pneumonia and ARDS, among military personnel in China, South Korea, and the United States [4–7], adenovirus-associated pneumonia has increasingly attracted the attention of researchers. Using whole-genome sequencing, prior studies identified more than 80 serotypes and suggested potential roles of some specific serotypes in different diseases and settings [8]. For instance, serotypes Adv-3, 7, 14, and 55 are implicated in acute respiratory disease [8–12], and Adv-7 and 55 could cause severe pneumonia [13,14].

Generally, doctors rely on clinical manifestations and chest computed tomography (CT) to diagnose adenovirus-associated pneumonia. The clinical and radiological features associated with this disease are nonspecific, that is, cough, fever, rhinorrhea, and dyspnea were the most common respiratory symptoms [15,16]. Laboratory examinations conducted in some studies indicated the presence of a relative lymphopenia and suppression of T-cell immunity in patients with adenovirus pneumonia, as demonstrated by low levels of CD3^+^, CD4^+^, CD8^+^, and CD20^+^ T-cells [1,12,17]. Adenovirus-associated pneumonia could be represented by imaging findings such as consolidation, ground-glass opacity, pleural effusion, septal thickening, and nodules [17,18], which are not specific enough to distinguish it from bacterial pneumonia.

Therefore, we aimed to compare clinical and imaging evidence between patients with adenovirus-positive (Adv+) pneumonia and Adv+ non-pneumonia and to investigate the association between cellular immunity and Adv+ pneumonia.

## 2. Materials and methods

### 2.1. Subjects

We retrospectively recruited 53 adult Adv+ patients who were admitted to Ruijin Hospital, Shanghai Jiao Tong University School of Medicine, from May 2015 to August 2019; among them, 30 and 23 patients had Adv+ pneumonia and Adv+ non-pneumonia, respectively.

The combination of the following 3 criteria was used to diagnose Adv+ pneumonia: (1) presence of respiratory symptoms (fever, cough, sputum, dyspnea, etc.), (2) presence of pulmonary infiltrates, consolidation, or ground-glass opacities on CT images, and (3) isolation of Adv from throat swabs. Adv+ non-pneumonia was defined by the isolation of Adv from throat swabs (Adv+), with no imaging evidence of respiratory symptoms and pneumonia. Viral co-infection was defined as positivity for two or more types of viruses in any combination. Bacterial co-infection was defined as positivity of other specimens (e.g., sputum, blood) for any kind of bacteria in combination with positivity for adenovirus.

Severe community-acquired pneumonia (CAP) was diagnosed according to the modified American Thoracic Society criteria [19,20]. Patients with shock requiring vasopressor therapy or mechanical ventilation or those fulfilling at least 3 of the following criteria were diagnosed with CAP: respiratory rate > 30 breaths/min at admission, PaO_2_/FiO_2_ ≤ 250, bilateral or multi-lobar involvement, white blood cell count ≤ 4000 cells/mm^3^, blood urea nitrogen level ≥ 20 mg/dL, core temperature < 36 °C, and hypotension with a need for aggressive fluid resuscitation.

The study was approved by the Ethics Committee of Ruijin Hospital, Shanghai Jiao Tong University School of Medicine.

### 2.2. Data collection

The following data were retrieved from the medical records of the subjects: demographic data (age, year, and duration of hospitalization), symptoms (fever, headache, cough, sputum, sore throat, tachypnea, chest pain, and chest tightness), underlying conditions (lung cancer, asthma, chronic obstructive pulmonary disease [COPD], pleural effusion, hypertension, hypoproteinemia, hepatic dysfunction, chronic kidney disease, glomerulonephritis, diabetes mellitus, and anti-neutrophil cytoplasmic antibodies [ANCA]-associated small vessel vasculitis), the use of immunosuppressive agents, CT findings, and laboratory examination results. The lymphocyte subset measurements were performed by flow Cytometry one day before or after the detection of adenovirus.

### 2.3. Detection of adenovirus and other pathogens

All throat swabs were examined for the presence of adenovirus and other respiratory viruses using multiplex real-time PCR. We screened for adenovirus, influenza A virus, influenza B virus, metapneumovirus, parainfluenza virus, rhinovirus, coronavirus, respiratory syncytial virus, bocavirus, and enterovirus. Nucleic acids were extracted using QiampMinelute Virus Spin Kit (Qiagen, Hilden, Germany). cDNA was synthesized and subjected to multiplex PCR using a respiratory virus 15-combination multiple PCR kit (Neuro-Hemin Biotech Co, Ltd, Hangzhou, China). An ABI 7500 (Applied Biosystems, California, USA) instrument was used to detect the viruses. The PCR reaction mixture contained 3 μL cDNA templates, 4 μL 5 × RV Primer, 3 μL 8-Mop Solution, and 10 μL 2 × multiplex master mix. The reaction mixture was subjected to the following steps: denaturation, 94 °C for 15 min; amplification, 40 cycles of PCR, 30 s at 94 °C, 90 s at 60 °C, and 90 s at 72 °C; and extension, 72 °C for 10 min. The 2^(−Delta Delta C(T)) (2^−ΔΔCT^) method was used for data analysis [21].

Blood, sputum, and urine samples were cultured to identify bacterial co-infections.

### 2.4. Statistical analysis

Statistical analysis was performed using GraphPad Prism 7 software. Data are shown as means ± standard deviation (SD) or median (IQR: 1st, 3rd quartiles) for continuous variables, and categorical variables were represented as numbers and percentages (n (%)). Data were compared using a two-sample t-test for continuous variables, and by chi-squared or Fisher’s exact test for categorical variables. A two-tailed p value < 0.05 was considered to be significant.

## 3. Results

### 3.1. Clinical characteristics

Clinical characteristics of the two groups are summarized in Table 1. Fifty-three patients tested positive for adenovirus, 30 of whom were diagnosed with pneumonia. The mean age of all the patients was 51.11 ± 21.88 years, and this study comprised 35 men (66.04%). Two patients with Adv+ pneumonia (6.7%) and 6 patients with Adv+ non-pneumonia (26.1%) used immunosuppressive agents. Twenty-five (83.3%) patients with Adv+ pneumonia had CAP. The duration of hospitalization of patients with Adv+ pneumonia (mean [IQR]: 16 [7, 29]) tended to be longer than that of patients with Adv+ non-pneumonia (8 [6, 11.5]) (p = 0.02). The top 3 frequent symptoms of Adv+ pneumonia were fever (66.7%), cough (63.3%), and tachypnea (16.7%). Patients with Adv+ pneumonia (19, 63%) were more likely to have cough and fever than were those with Adv+ non-pneumonia (4, 17.4%) (p = 0.001). Patients with chronic kidney disease were more common in the Adv+ non-pneumonia group than in the Adv+ pneumonia group (14, 60.9% vs. 3, 10%) (p < 0.001). There were no significant differences in other clinical characteristics between the two groups (p > 0.05).

### 3.2. Chest CT findings

**Figure 1:**
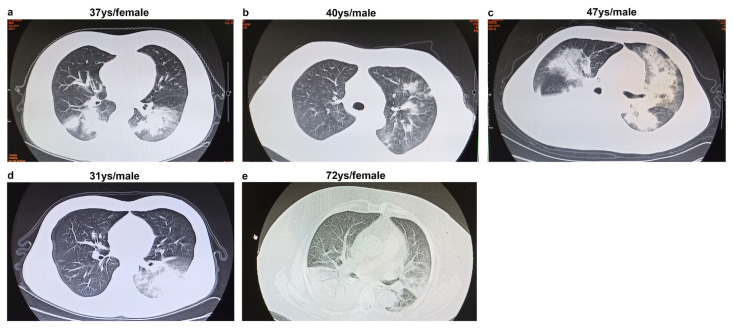
(a–e) Computed tomography images of five patients with adenoviral pneumonia showing consolidation and surrounding ground-grass opacity.

Chest CT findings are presented in Table 2. Chest CT findings of patients with Adv+ non-pneumonia were different from those of patients with Adv+ pneumonia (Figures 1a–1e). Most of the patients with Adv+ pneumonia were diagnosed with CAP (25, 83.3%). CT features of the Adv+ pneumonia group were as follows: consolidation, 22 patients (73.3%); ground-glass opacities, 8 patients (26.7%); spot shadows, 7 patients (23.3%); nodular lesions, 5 patients (16.7%); and pleural effusion, 6 patients (20%). Notably, 5 patients (16.7%) showed classical CT findings (Figure 1): conspicuous consolidation with surrounding ground-glass opacity. The lesions of pneumonia were more likely to involve bilateral lobes (18, 60%), followed by the right lobe (10, 33.3%) and the left lobe (2, 6.7%).

### 3.3. Laboratory examinations

Comparative findings of laboratory examinations between the two groups are displayed in Table 3. In patients with Adv+ pneumonia, the white blood cell (WBC) count, neutrophils percentage, and C-reactive protein (CRP) level were 7.1 ± 3.9 × 10^9^/L, 70.66% ± 13.58%, and 7.92 (2.25, 66.75) (mean [IQR]) g/L, respectively, and 82.6% of patients showed a Procalcitonin (PCT) level of <0.5 ng/mL. Patients with Adv+ pneumonia showed higher levels of blood glucose [mean [IQR], 6.62 (5.43, 11.05) mmol/L vs. 4.7 (4.22, 5.67) mmol/L, p = 0.001] and lower levels of albumin (p = 0.037) and uric acid (p = 0.008) than did those with Adv+ non-pneumonia. Few patients in both the groups showed evidence of viral or bacterial co-infection. For instance, in Adv+ pneumonia patients (n = 30), 2 (6.7%) patients were co-infected with bacteria (Klebsiella pnuemoniae and Staphylococcus aureus, respectively). In Adv+ non-pneumonia patients (n = 23), one patient was co-infected with Klebsiella pnuemoniae, and 3 patients were co-infected with viruses, including Respiratory syncytial virus (n = 2) and influenza B virus (n = 1).

### 3.4. White blood cell immunophenotypes

We also compared the white blood cell immunophenotypes in patients with Adv+ pneumonia (n = 21) and Adv+ non-pneumonia (n = 16) (Table 4). Patients with Adv+ pneumonia showed significantly lower numbers of CD3^+^ (p = 0.034), CD4^+^ (p = 0.031), and CD8^+^ T-cells (p < 0.004) than did those with Adv+ non-pneumonia.

**Figure 2:**
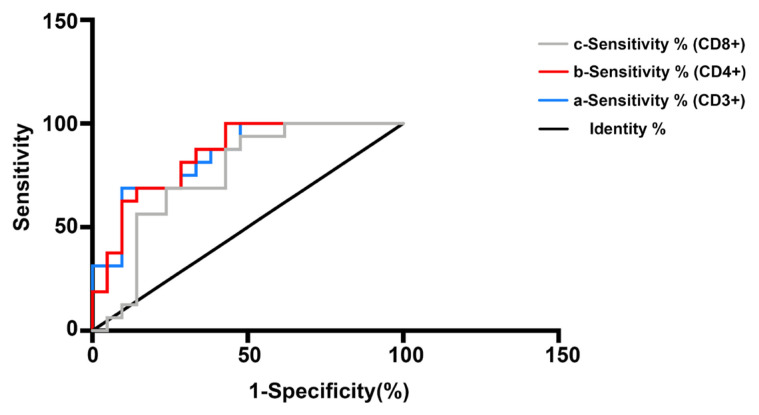
Receiver operating characteristic (ROC) curve of absolute number of CD3+, CD4+, CD8+ T cells to evaluate for the presence of pneumonia in in Adv+ patients. The areas under the ROC curve (AUC) of counts of CD3+ (a), CD4+ (b), CD8+ (c) T cells to predict severity of the disease were 0.8423, 0.8482, and 0.75 respectively.

We further analyzed the receiver operating characteristic (ROC) curve to analyze the accuracy of the counts of CD3^+^, CD4^+^, and CD8^+^ T-cells for use as a biomarker of pneumonia in Adv+ patients. Areas under the ROC curve for CD3^+^, CD4^+^, and CD8^+^ T-cells were 0.8423 (Figure 2a), 0.8482 (Figure 2b), and 0.75 (Figure 2c), respectively, indicating that counts of CD3+, CD4+, and CD8+ T-cells may be used as predictors of pneumonia in Adv+ patients.

## 4. Discussion

The present retrospective study, which compared the clinical features, image findings, and laboratory results between patients with Adv+ pneumonia and Adv+ non-pneumonia, showed that Adv+ pneumonia was associated with a higher risk of cough and fever, longer duration of hospitalization, higher glucose level, and lower albumin and uric acid levels. Consolidation was the most common imaging chest CT finding of patients with Adv+ pneumonia, who were also revealed to harbor lower numbers of CD3+, CD4+, and CD8+ T-cells.

Adenovirus was initially identified in young military recruits in the United States [22]. Over the following decades, multiple outbreaks of adenovirus-related respiratory illness in different populations shed light on the self-limiting course of adenovirus-induced infections and mild symptoms in a majority of immunocompetent individuals [23,24]. The infections that progressed with fatal courses (CAP or ARDS), requiring intensive care and invasive trachea intubation, and resulting in fatal outcomes, have been more commonly reported in immunocompromised patients than in immunocompetent patients [1–3]. In our study, severe Adv+ pneumonia was observed in 3 (10%) immunocompetent patients.

Previous reports have identified the most common symptoms of adenovirus infections in the United States military recruits as fever and cough [7,25]. A retrospective study involving 80 hospitalized children with adenovirus-associated pneumonia in Taiwan, conducted from 2000 to 2008, reported cough (99%), fever (96%), rhinorrhea (82%), dyspnea (42%), gastrointestinal symptoms (vomiting, diarrhea, and abdominal pain), and neurological symptoms as the most common symptoms [15]. Among Korean military personnel, the most frequently observed symptoms were cough (83%–98%) and fever (83%–98%); diarrhea was seen only rarely (12%–20%) [16,26]. Moreover, reportedly, adenovirus-associated pneumonia was significantly associated with a higher risk of fever, high fever (> 39 °C), nasal congestion, sore throat, throat clearing, headache, and pharyngeal inflammation than was adenovirus-negative pneumonia [26]. However, in the present study, Adv+ pneumonia manifested with fever (66.7%), cough (63.3%), and tachypnea (16.7%), and Adv+ pneumonia was correlated with a higher risk of fever and cough and longer duration of hospitalization than was Adv+ non-pneumonia. These results were in agreement with previously published results, except for the prevalence rate of fever. This may be explained by a relatively higher incidence rate of bacterial co-infection (confirmed by PCR test and Gram staining/microbial culture) in the previous study [16] than in the current study.

A previous study revealed the WBC (8.3 × 1000 cells/μL) and neutrophil counts (6.7×1000 cells/μL) of United States military trainees with Adv-14 pneumonia [27]. In the current study, similar results were observed in the Adv+ pneumonia group: WBC counts, 7.1 ± 3.9 × 10^9^/L) and percentage of neutrophils (70.66% ± 13.58%); however, these data were not significantly different between patients with Adv+ non-pneumonia and Adv+ pneumonia. Patients with Adv+ pneumonia showed elevated CRP levels and PCT levels < 0.05 ng/ml; however, 5 patients (16.7%) showed elevated PCT levels (> 0.5 ng/mL). Moreover, only 2 patients with Adv+ pneumonia (6.7%) showed evidence of bacterial co-infection: Klebsiella pnuemoniae and Staphylococcus aureus, respectively. The rarity of bacterial and adenovirus co-infection led us to propose the presence of AdV-associated pneumonia in our patients.

Previous studies evaluated a wide spectrum of CT images from different sample populations and found the following features in different proportions: consolidation, ground-glass opacity, pleural effusion, septal thickening, and nodules; however, till date, there is no consensus regarding the radiological manifestations of Adv+ pneumonia. Chan et al. evaluated the CT images of 104 immunocompetent patients with Adv+ pneumonia and reported that the most frequent pattern was consolidation with or without surrounding ground-glass opacity, with subpleural and peribronchovascular distributions [18], a pattern that was also observed in the CT images of 152 Korean military personnel with Adv (consolidation with ground-glass opacity patterns showing lobar distribution) [16]. Moreover, ground-glass opacity was observed more frequently in patients with adenovirus infection than in those with other viral and bacterial infections [28]. Patients with Adv+ pneumonia in the present study were more likely to show consolidation; however, only 8 patients (26.7%) showed ground-glass opacities and only 5 immunocompetent patients showed the previously mentioned traits of conspicuous consolidation with surrounding ground-glass opacity. Some studies showed that unilateral involvement or single-lobe involvement was more common in patients with Adv+ pneumonia [18,26], which contradicts the finding of the present study (bilateral lesions) [29,30]. However, large-scale imaging findings on adenovirus pneumonia are necessary to confirm the CT features suggestive of this disease at the time of presentation.

Immune responses play an essential role in adenovirus infection, which may also explain why individuals with immunodeficiency are more susceptible to severe Adv+ pneumonia. Host response to adenovirus infection has been observed in humans and animal models. Adenovirus initially triggers a robust innate immune response, causing a wide spectrum of viral-induced responses via cellular pathways [31,32]. Chen et al. reported that severe Adv-55 infection was significantly associated with excessive immune reactions or inflammatory responses such as increases in serum IFN-γ, IL-4, and IL-10 levels [14]. Another study involving 21 cases of adenovirus pneumonia reported relative lymphopenia [1]. A retrospective study conducted in Taiwan indicated an inhibition of cellular immunity, as evidenced by significant reduction in counts of CD4^+^, CD8^+^, and CD20^+^ T-cells in patients with Adv-7 pneumonia showing pleural effusion [17]. Toth et al. demonstrated a significant decrease in populations of CD3^+^, CD4^+^, and CD8^+^ T-cells in the liver and spleen of Syrian primates at 1 and 7 days after intravenous injection of adenovirus [33]. Similarly, our data implied greater cellular immunosuppression in patients with Adv+ pneumonia, as demonstrated by a decline in counts of CD3^+^, CD4^+^, and CD8^+^ T-cells, than in patients with Adv+ non-pneumonia. Even though further analysis of the ROC curve suggested that counts of CD3^+^, CD4^+^, and CD8^+^ T-cells may be used as predictors of pneumonia in Adv+ patients when combined with CT findings and clinical symptoms, the results should be interpreted with caution and need further research in the light of many factors that may contribute to this condition.

There are some limitations in present study that warrant mentioning. Firstly, the small sample size of this study precluded our ability to further subgroup analysis. For instance, in a large-scale study, the comparisons of clinical and radiological features between immunosuppressed and immunocompetent patients with Adv+ pneumonia or Adv+ non-pneumonia could shed light upon some clinical implications toward understanding the pathogenesis of the disease. Secondly, the retrospective search excluded some AdV+ patients in our study, as PCR tests for respiratory viruses was not routinely performed on all patients. This is also a bias towards eligible cases in this study. Lastly, in our setting, we did not determine the adenovirus serotypes, which may provide some clues in the observed differences of clinical and imaging findings between patients with Adv+ pneumonia and Adv+ non-pneumonia. Moreover, clinical presentation, severity and outcomes may vary from AdV types.

In conclusion, our data showed that the frequently observed symptoms of Adv+ pneumonia are cough and fever, and that the most common image findings were consolidation; however, these results were not specific for Adv+ pneumonia. We also demonstrated that cellular immunity is compromised in patients with Adv+ pneumonia.

## Figures and Tables

**Table 1:** Clinical characteristics of patients with Adv+ pneumonia and Adv+ non-pneumonia (n = 53).

Variables	Total (n=53)	Adv+ pneumonia (n = 30)	Adv+ non-pneumonia (n = 23)	P value	KS normality test	
					p value*	p value#
Age, year	51.11 ± 21.88	46.33 ± 15.16	60.67 ± 28.97	0.531	0.072	0.094
Sex (male)	35 (66.04%)	19 (63.3%)	16 (69.6)	0.772	-	-
Duration of hospitalization (days), median (IQR)	11 (7, 23)	16 (7, 29)	8 (6,11.5)	**0.029**	0.0019	0.0028
Time from admission to detection of Adv, day, median (IQR)	3 (2,4)	3 (1.25,5.25)	3 (2,4.5)	0.62	<0.0001	0.0039
Symptoms						
Fever (≥ 38°C)	21 (39.6%)	20 (66.7%)	1 (4.3%)	**<0.001**		
Headache	2 (3.8%)	1 (3.3%)	1 (4.3%)	1	**-**	**-**
Cough	23 (43.4%)	19 (63.3%)	4 (17.4%)	**0.001**	-	-
Sore throat	1 (1.9%)	1 (3.3%)	0	1	**-**	**-**
Tachypnea	5 (9.4%)	5 (16.7%)	0	0.061	-	-
Chest pain	1 (1.9%)	1 (3.3%)	0	1	**-**	**-**
Chest tightness	3 (5.7%)	2 (6.7%)	1 (4.3%)	1	**-**	**-**
CAP (%)	25 (47.2%)	25 (83.3%)	-	-	**-**	**-**
Severe pneumonia	3 (5.7%)	3 (10%)	-	-	-	-
Use of immunosuppressive agent (Corticosteroid)	8 (15.1%)	2 (6.7%)	6 (26.1%)	0.065	**-**	**-**
Underlying condition					-	-
Lung cancer	4 (7.5%)	1 (3.3%)	3 (13%)	0.624		
Asthma	3 (5.7%)	2 (6.7%)	1 (4.3%)	1	**-**	**-**
COPD	3 (5.7%)	2 (6.7%)	1 (4.3%)	1	-	-
pleural effusion	9 (17.0)	6 (20%)	3 (13%)	0.715	**-**	**-**
Hypertension	23 (43.4%)	11 (36.7%)	12 (52.2%)	0.280	-	-
Hypoproteinemia	3 (5.7%)	3 (10%)	0	0.249	**-**	**-**
Hepatic dysfunction	13 (24.5%)	8 (26.7%)	5 (21.7%)	0.749	**-**	**-**
Chronic kidney disease	17 (32.1%)	3 (10%)	14 (60.9%)	**<0.001**	**-**	**-**
Glomerulonephritis	6 (11.3%)	2 (6.7%)	4 (17.4%)	0.385	**-**	**-**
Diabetes mellitus	12 (22.6%)	6 (20%)	6 (26.1%)	0.743	-	-
ANCA-associated small vessel vasculitis	2 (3.8%)	1 (3.3%)	1 (4.3%)	1	**-**	**-**

IQR=interquartile range, COPD=Chronic Obstructive Pulmonary Disease, CAP=Community acquired pneumonia. P value* means the p value of Shapiro-Wilk normality test in Adv+ pneumonia group.

P value* means the p value of KS normality test in Adv+ pneumonia group, P value# means the p value of KS normality test in Adv+ non-pneumonia group.

Data are shown as mean ± SD, median (IQR) or number (%).

**Table 2:** Characteristics of chest CT findings in patients with Adv+ pneumonia (n = 30).

Characteristics	Adv+ pneumonia (n = 30)
Pattern	
Consolidation	22 (73.3%)
Ground-glass opacity	8 (26.7%)
Spot shadow	7 (23.3%)
Nodular lesions	5 (16.7%)
Consolidation and ground-glass opacity	5 (16.7%)
Pleural effusion	6 (20%)
Mainly involved lobe	
Only left lobe	2 (6.7%)
Only right lobe	10 (33.3%)
Either lower lobe	12 (40%)
Bilateral lobes	18 (60%)

Data are shown as number (%).

**Table 3:** Laboratory examinations between patients with Adv+ pneumonia and Adv+ non-pneumonia (n = 53).

Laboratory Data	Total (n = 53)	Adv+ pneumonia (n = 30)	Adv+ non-pneumonia (n = 23)	p value	KS normality test	
					P value*	P value#
WBC, 10^9^/L	7.29 ± 3.22	7.1 ± 3.9	7.53 ± 1.96	0.647	>0.1	>0.1
Neutrophil (%)	68.32 ± 11.63	70.66 ± 13.58	65.27 ± 7.38	0.098	>0.1	>0.1
Lymphocyte (%)	20.96 ± 10.67	19.65 ± 12.38	22.66 ± 7.56	0.318	>0.1	>0.1
CRP (g/L) (n=44), median (IQR)	7.39 (1.34, 44)	7.92 (2.25, 66.75)	2.83 (1, 35.15)	0.220	<0.0001	0.0003
PCT (<0.5ng/ml) (n=39)	0.05 (0.05, 0.16)	0.05 (0.05, 0.35)	0.05 (0.05, 0.1)	0.639	<0.0001	<0.0001
ESR, mm/h (n=32)	26.49 ± 24.11	34.98 ± 69.48	19.89 ± 20.30	0.083	>0.1	0.06
Albumin (g/L)	33 (28, 35)	29 (27, 34)	35 (32, 36)	**0.037**	>0.1	0.038
ALT (IU/L), median (IQR)	22 (15, 45)	26 (17, 69.5)	20 (15, 39)	0.161	<0.0001	0.0003
AST(IU/L), median (IQR)	25 (19, 46)	32.5 (20.25, 62.75)	21 (18, 34)	0.145	<0.0001	<0.0001
Blood glucose (mmol/L)	5.56 (4.62, 7.29)	6.62 (5.43, 11.05)	4.7 (4.22, 5.67)	**0.001**	0.0277	>0.1
Creatinine (μmol/L)	78 (57.5, 111)	51 (8.84, 78.5)	76.8 (38.4, 117)	0.425	<0.0001	<0.0001
Uric acid (μmol/L)	234 (174.5, 379.5)	199.5 (146.8, 269.5)	314 (239, 419)	**0.008**	<0.0001	>0.1
BUN (mmol/L)	5.4 (4, 8.1)	5.15 (3.65, 6.6)	5.5 (4.3, 9.9)	0.701	<0.0001	<0.0001
Viral co-infection (%)	3 (5.7%)	0	3 (13%)	0.075	-	-
Bacterial co-infection (%)	3 (5.7%)	2 (6.7%)	1 (4.3%)	1	-	-

WBC=White blood cell, CRP=C-reactive protein, ESR= Erythrocyte sedimentation rate, ALT=Alanine aminotransferase, AST=Aspartate aminotransferase, BUN=Blood urea nitrogen.

P value* means the p value of KS normality test in Adv+ pneumonia group, P value# means the p value of KS normality test in Adv+ non-pneumonia group.

Data are shown as mean ± SD, median (IQR) or number (%).

**Table 4:** Immune phenotype of white blood cells of patients with Adv+ pneumonia and Adv+ non-pneumonia (n=39)

variables	Adv+ pneumonia (n = 21)	Adv+ non-pneumonia (n = 16)	p value	KS normality test	
				p value*	p value#
CD3+ T cells, cells/mm3	346.5 (204, 1259)	1851 (1109, 2012)	< **0.001**	0.0091	0.0833
CD4+ T cells, cells/mm3	217 (102.2, 734.8)	975 (604, 1346)	< **0.001**	0.0087	>0.1
CD8+ T cells, cells/mm3	152 (64.25, 403.3)	570 (382, 764)	**0.03**	0.002	>0.1

P value* means the p value of KS normality test in Adv+ pneumonia group, P value# means the p value of KS normality test in Adv+ non-pneumonia group.

Data are shown as median (IQR).

## Data Availability

All data generated or analyzed during this study are included in this published article.
